# (Hidden) potentials for African languages in curriculum reforms: examples from Kenya and South Africa

**DOI:** 10.1007/s43545-022-00440-6

**Published:** 2022-08-05

**Authors:** Michael M. Kretzer, Everlyn Oluoch-Suleh

**Affiliations:** 1 Department of Human Geography, Ruhr Universtät Bochum, Bochum, Germany; 2 Department of African Language Studies, University of Western Cape (UWC), Cape Town, South Africa; 3 School of Humanities and Social Sciences, United States International University - Africa, Nairobi, Kenya

**Keywords:** Kenya, South Africa, Competency-Based Curriculum (CBC), African languages, Kiswahili, Outcomes-Based Education (OBE)

## Abstract

Education systems are globally reformed to focus more on competencies and be more pupil-centred. Post-colonial countries like Kenya and South Africa face severe educational challenges regarding access, language policy and the quality of education. Both countries share a colonial history under the British Empire. South Africa rolled out its Outcomes-Based Education (OBE) curriculum, but soon reformed and later changed it substantially to the new Curriculum and Assessment Policy Statement (CAPS). Kenya implemented their Basic Education Curriculum Framework (BECF) only recently in 2017, which represents a Competency-Based Curriculum (CBC). Both curricula do not have language policy as a priority, although many children in both countries have very limited exposure and competencies in English, the dominant language of learning and teaching (LoLT) in Kenya and South Africa. They can read English words in lessons, but quite often cannot explain their meaning. A semi-systematic literature review was conducted to analyse common or similar and different patterns in both countries as well as the academic representation of it. Together with own previous research, the study revealed that ideally language policy and curricular reforms need to be addressed simultaneously.

## Introduction

Education with an unfamiliar and even (partly) unknown language is hindering cognition in many countries of the global south and some authors describe it as torture (Kioko et al. 2014, p. 2). They use the following metaphor to illustrate teaching in many (rural) schools in Africa.

Expecting them to write and read effectively what they do not speak or understand is like asking them to run when they have not learned how to stand alone! It is like plunging the young learners without swimming skills into a fast-flowing river and expecting them to make it to the other side where the teacher and the whole school system will compliment them for their success. Experience has shown that the few lucky ones get across, but the majority of them sink. (Kioko et al. 2014, p. 3).

During the 1990s educational efforts increased globally. Countries of the global south struggled significantly with low and uneven distributed enrolment. The UN formulated Sustainable Development Goals (SDGs), which concentrate on qualitative aspects of education. In specific SDG 4 with its sub-targets aimed at improving and measuring the quality of education. Sub-target 4.5 focuses on the elimination of existing disparities between gender and indigenous communities. Indicator 4.5.2 gives the percentage of pupils at primary schools being taught in their First Language (L1) (UNESCO 2018a).

Such international efforts had significant effects on education systems in African countries. Not only its structures were modified or training of teachers, but rather many curricular reforms evolved. Language as a key element and variable of education was highlighted by UNESCO in its 1953 report ‘The use of vernacular languages in education’ (UNESCO 1953). A corpus of literature and reports by UNESCO and others showed the disadvantage of the selection of the colonial language. Around 40% of the global population received or receive education in an unfamiliar language. Despite research indicating the benefits of using the pupil's First Language (L1) as Language of Learning and Teaching (LoLT), it is largely ignored. First, language policies are often changed and vaguely formulated. Second, an early-exit model with a shift to English as LoLT at grade 3 or 4, regardless of socio-cultural, linguistic circumstances or the (official) language policy is often in place. At some schools, teachers use English from grade 1 as LoLT (Trudell 2016). The main problem lies in the somehow artificial, but deeply rooted dichotomy of either using English as LoLT or an African language. If an African language is used as LoLT then it is only as a bridge to English. Such a devalued view of African languages remains an obstacle. Often teachers and parents see African languages as inappropriate for meaningful formal education and prefer the learning of English through ‘submersion’ as a most ‘effective’ way of teaching. Such language attitudes favour English and make use of African languages as LoLT nearly impossible (Trudell and Piper 2014, p. 10). Recent curriculum reforms put children at the centre of lessons. Hence, the use of an appropriate language to enhance meaningful teaching and learning should go hand in hand with new curricula, which include a child-centred pedagogy and aspects of constructivism.

The study tries to answer the following research question: 1. How are teachers in Kenya and South Africa involved in the process of language policy and curricular formulations? 2. What is the role of English, Kiswahili and other African languages in language policies and curricula in both countries?

Kenya and South Africa were selected to highlight certain similar patterns in language policy developments for the vast majority of African countries. The role of Kiswahili in Kenya and the selection of Kiswahili as an optional subject in South Africa also connect both multilingual countries. Furthermore, both countries are former British colonies or heavily influenced by English settlers and the English language historically as well as during globalization in recent times. Both countries formulated a high variety of language policy documents and changed their language policy after independence several times as well as undertook many educational changes and curricula reforms in the last decades. Significant differences exist between both countries as shown below, e.g. the historical legacy of apartheid, and the different role or status of African languages in both countries among others.

The study is structured in six parts. The first one gives an overview of the methodology, followed by a conceptual section, which includes definitions and historical placement of Competency-Based Education (CBE) or Competency-Based Curriculum (CBC) in education systems of the global south. Thirdly, the socio-linguistic, as well as administrative structures of Kenya and South Africa, are described. The fourth part is devoted to historical and political circumstances. In the fifth part, the recent curricula are analysed and compared with a specific focus on language policy and the status of indigenous languages. This section includes some findings of previously conducted fieldwork in South Africa by one of the authors, followed by a conclusion.

## Methodology

This study used a semi-systematic literature review. The reasoning to conduct such a methodological approach was twofold. Firstly, a pure systematic literature review on such a broad topic, covering diverse disciplines as well as using different methodologies seemed to be rather inappropriate. Contrary, a semi-systematic review offers the opportunity to identify and describe existing research topics or themes as well as applied concepts or methodologies. Secondly, reviewing all published articles concerning this topic is just not possible at all. This semi-structured review aims to identify and understand all potential relevant research traditions that have implications for the studied topic and to synthesize these using meta-narratives (Snyder 2019, p. 335).

Hence, this study analysed academic publications about both research countries mainly over the last two decades or so. It includes those available online and in print. Recent curriculum developments were analysed as well as various policy papers and educational reforms. Publications in English and German were included and were found by using mainly Google Scholar and library databases. To give examples, some keywords were, among many others, ‘language policy in South Africa’, language policy in Kenya’ ‘Curriculum in Kenya’. Both countries have a long history of changing language policies and curricula. Therefore, isolated publications from the previous century were considered, in case such ones remain key articles up until today. To understand and analyse the recent curricula of both countries historical developments were researched. As one of the authors worked in South Africa and did extensive fieldwork some of his previous collected insights will be used. Previous fieldwork in South Africa included semi-structured interviews with over 25 educational stakeholders as well as an analysis of vast quantitative data focusing predominantly on language attitudes and language practices of primary and secondary school teachers. Nevertheless, the study emphasizes literature review and analysis of political documents. In the following section, the definition or understanding of CBC and its origins are described more in detail.

## Origins, development and spread of CBC in sub-saharan Africa

After the independence of African states, the focus was on educational expansion. Due to limited access to education, the vast majority of African governments tried to increase the capacity of their institutions. After producing textbooks, increasing access to universities and building schools the focus shifted to teacher education as their qualifications remained low. Up until the 1990s, the focus was on equal access to education. This was and remains important. Due to the COVID-19 pandemic and widespread closure of schools, excluding millions of pupils, it is again very important. As mentioned above the SDG focused predominantly on quality education. The question was no longer IF a child goes to school, but rather HOW and WHAT pupils learn at schools. Teaching should be meaningful for pupils. Therefore, curricular and pedagogical reforms became more and more necessary and urgent. Nearly all African lessons were /(are) teacher-focused, a former colonial language was used and merely rote learning happens, often represented by choir answers in the classroom. Such lessons restrict the activities of pupils and they are rather passive recipients of information and chalkboard notes. Their role is limited to memorizing facts, so a clear focus on content becomes visible. Contrary CBC allows much more pupil activities and aims to strengthen critical thinking and social competencies. Hence, CBE formulates Curricula backwards from the needed skills (Gervais 2016, p. 100). A clear paradigm shift from content to competencies and outcomes is the core, which is also often described as child-centred pedagogy (CCP). Those curricula are based on constructivist principles. The focus of constructivism is also often criticized due to its limitations. For example, if constructivist principles have been used for teaching and learning in rural, townships or other disadvantaged learning environments the connectivity to lived experiences of the pupils is rather limited. Constructivist principles' inability or difficulties in transforming such pupils' experiences of other socio-economic milieus to overcome existing socio-economic inequalities are often highlighted (Akala 2021, p. 2). Such teaching changes not only the pedagogical approach but rather the role of teachers and pupils and the classroom organizations in general. According to constructivist ideas, the best and most successful learning happens when it can be connected or built on the already existing knowledge of pupils. Therefore, teaching is relevant and connectable to pupils' prior life experiences.

There is no uniform definition of CBC or Competency-Based Education (CBE) (Burnette 2016, p. 85) as the overarching term, focusing on the entire education and not only the curricula in place.

It is rather defined in heterogeneous and multiple ways and its interpretation and implementation vary over time and across various academic programmes. Often CBE is used and researched in vocational training contexts as well as in medical education. A variety of other terms hinder a uniform definition as CBE is sometimes phrased as OBE, problem-based learning or performance-based learning, just to name a few. Nevertheless, CBE is the accepted and commonly used overarching term and the formal terminology. Overall, academics describe CBE often as an ‘eclectic model adopting concepts from several modern learning theorists’ (Gervais 2016, p. 99). Underlying theoretical or conceptual frameworks include behaviourists, functionalists as well as humanistic ones. Such theorists include Ralph W. Tyler (1976) and his focus on dynamism and student-centred approach remains the foundational basis of CBE. Based on such Fred Keller emphasized self-paced learning and adds to the conceptual grounding of CBE. Therefore, CBE influences learning pace, way of instruction and teaching and the entire way of assessment, so basically the entire school culture.

Two early definitions capture the above-mentioned origins as well as the underlying theoretical foundational kaleidoscope of CBE given by Spady (1977) and Riesman (1979). Spady emphasizes in his definition of CBE aspects such as “a data-based, adaptive, performance-oriented set of integrated processes that facilitate, measure, record and certify within the context of flexible time parameters the demonstration of known, explicitly stated, and agreed upon learning outcomes that reflect successful functioning in life roles” (Spady 1977, p. 10). Riesman adds to it the above-mentioned aspect of its roots when he states that CBE derives "from an analysis of a prospective or actual role in modern society” and formulates the ‘flexible time parameters’ (Spady 1977, p. 10) much more critical. “Theoretically, such demonstrations of competence are independent of time served in formal educational settings” (Riesman 1979, p. 6).

Some authors see the Morrill Lands Act of 1862 as the first approach for applied education to prepare now pupils to become doers instead of thinkers (Gervais 2016, p. 99). Overall, CBE exists as an educational approach since the 1970s and gained increasingly global interest in the 1990s. As mentioned above, some characteristics or aspects are essential for CBE and CBC, such as focusing on ‘learning outcomes’ or ‘competencies’ as well as flexibility for pupils to master certain skills at their own pace. Hence, traditional concepts like credit hours or teacher-centred traditional classrooms are not part of it. Therefore, the traditional year-end examinations are also a relic of the past and CBE uses directly ongoing assessments. Literature indicated some major challenges with the CBE concept. Those challenges are even larger for resource-limited schools to be able to offer individual teaching for different learning paces of pupils. A very general critique comes from Stephanie Allais about competency bases National Qualifications Framework as social constructivism and neoliberalism in her book 'Selling out Education'. For her CBE entails significant flaws, especially of not so well-resourced education systems trying to implement it (Allais 2014). In this regard, Blackmur (2015) criticized her book as very detailed due to poor explications or numerous faulty or at least unproven causal relationships done by her. CBE-focused teaching lessons should be structured in a participatory way, which allows group or partner work so that the classroom interaction is active and democratic (Altinyelken 2010, pp. 152–153). Such a new approach to teaching also prefers continuous and various forms of assessment, instead of year or term-end examinations. Some critics see CBC as a post-colonial approach, because of its 'Western' origins. Most of the time the reformed CBC in African countries represent only a slight modification of its 'Western’ roots (Ruth and Ramadas 2019, p. 47). A significant gap between the official policy and its actual daily implementation however exists. If teachers are not guided and trained in the development and formulation of a new CBC, implementation failure is a likely outcome. Education systems are quite conservative and change slowly. Behaviours, own experiences and long-lasting learning and teaching under a teacher-centred curriculum make CBC reforms challenging.

These case studies seem to suggest that prescriptive instructional behaviour is so deeply embedded in the professional culture that even if child-centred approaches are initially embraced, they disappear with time and are replaced by traditional instructional behaviour (Altinyelken 2010, p. 156).

In general, implementation remains a challenge, and often a kind of hybrid, between the previous, traditional curriculum with its underlying ideology and the new curriculum, that are been implemented. Kagema (2018) highlights that successful implementation of curricula is only possible if teachers are involved, trained and have knowledge about it. Only then can their anxiety about knowledge change be minimized. The next section describes the socio-linguistic settings of the Kenyan and South African education systems.

## Socio-linguistic, administrative and constitutional context of Kenya and South Africa

Kenya and South Africa are both multilingual and heterogeneous countries in Sub-Saharan Africa. For both countries, a huge variation exists regarding the number of languages. They were both colonized and are categorized as Anglophone^1^. Kenya has around 40 indigenous languages (Mwaniki 2014, p. 11). Some authors such as Mandillah (2019, p. 2) mention a significantly higher number when she speaks of over 70 different languages and language varieties. Similarly, Githiora (2002, p. 162) mentions over 60 languages and language varieties. The majority mention 42 (Ogechi 2009, p. 143; Ogechi and Bosire-Ogechi 2002, p. 168) and others ‘acknowledged’ 44 (Spernes and Ruto-Korir 2018, p. 42) languages. Some are not so precise and see more than forty (Mose 2017, p. 215) or indicate 42 (Kibui 2014, p. 89; Ogechi 2003, p. 279), but reference a report by the Kenyan government, which mentions 70 languages. The Constitution of Kenya Review Commission mentions ‘70 ethnic communities clustered into at least 42 groups’ (Kenya Law 2005, p. 58) but clearly states at a later stage of the report: 'There are over 90 languages in Kenya spoken by over 90 distinct ethnic, sub-ethnic and foreign linguistic groups, which have their traditions, customs and history' (Kenya Law 2005, p. 95) and refers also to the ‘42’ as not representative for Kenya.

Ethnologue counts 68 languages in Kenya (Ethnologue 2016a). This overview shows the multilingual situation, but also indicates the difficulty of how to 'name' and 'categorize' certain language varieties as distinct languages or 'dialects'. The situation gets more complicated with recent language developments such as 'Sheng'. Sheng is a mixed language of Swahili and English, which originates from the highly multilingual situation of the urban youth in Nairobi (Githiora 2002).

The number of languages in South Africa also varies depending on different sources. Ethnologue (2016b)` counts 34, whereas Kamwangamalu (2001, p. 363) or the Department of Arts and Culture (2003: 5) only lists 25. Similar to the situation in Kenya a variety of pidgin or creole languages have developed. The situation gets further complicated by the fact that indigenous languages exist on a continuum with many slightly different language varieties. Kaschula and Kretzer (2019) analysed the effects of language varieties of isiXhosa on the teaching of Standard-isiXhosa in two different South African provinces and its challenges.

Similar to Kenya languages in South Africa are regionally concentrated. Rural South Africa is mainly homogenous and only urban or peri-urban areas are heterogeneous and multilingual. South Africa consists of nine provinces and 52 districts. Language policy is not limited to the national layer, but rather on the micro-level of the South African government and its associated institutions. Kenya's new Constitution of 2010 also aims for decentralization. Kenya consists of 47 counties as stipulated under Article 6 (Kenya Law 2010). Nevertheless, both countries have a central education system with one central curriculum and no de-centralized curricula. Due to devolution policies, a more flexible approach at schools is in both countries possible.

Art. 7 declares Kiswahili a national language and both English and Kiswahili official languages. Furthermore, constitutional recognition of multilingualism happened for the first time in Kenya's history.

‘(3) The State shall -- (a) promote and protect the diversity of language of the people of Kenya; and (b) promote the development and use of indigenous languages, Kenyan Sign language, Braille’ (Kenya Law 2010: Art. 7(3)).

Hence, Kiswahili is the only directly named indigenous language next to the Kenyan Sign language in the Kenyan Constitution.

Contrary, the Constitution of South Africa, 1996, declares in Art. 6(1) eleven languages official (Republic of South Africa 1996) and directly named nine indigenous languages. Such a progressive multilingual approach is unique globally. Under Article 6(5) further languages are mentioned to be developed and promoted such as the Sign Language or the Khoe-San language cluster, but even historical migrant languages like German or Hindi and others are protected. This progressive character is limited through formulations like ‘be treated equitably’ in Article 6(4). The vague formulations hinder a clear implementation of language policy and led to many court cases (Malan 2011, p. 392). Nevertheless, such status planning remains progressive and inclusive from a legal perspective. In the following section, the underlying historical and socio-cultural developments in both countries are described.

## Historical and socio-cultural developments of the education systems and curricula in Kenya and South Africa

### African languages and publications

South Africa has a long history of publications in indigenous languages, which can be traced back to the 19th century. Examples are Sol Plaatje’s publication *Tsala ea Batho* (The friend of the people) in Setswana and the 1884 published isiXhosa newspaper *Imvo Zabantsundu* (African opinion) by Jabavu. Motsaathebe (2011, p. 121) offers a list of publishing houses to indicate the limited, but the existent market for indigenous language publishing. Overall the indigenous book market in South Africa depends heavily on the education sector due to the socio-economic situation in the country.

Some authors see 1850 as the earliest time for Kiswahili publications in Kenya (Campbell and Walsh 2009, p. 580), which is even some decades ahead of South Africa. Ogechi and Bosire-Ogechi (2002, p. 171) listed around 130 registered publishing houses in Kenya, which also depend largely on the school market.

Therefore, both countries have a small, but working book market, including publications in various African languages. Additionally, they have a long history of publications in African languages and many are standardized and used within the education system. Both book markets depend significantly on decisions and developments of the education system, because of a very limited non-educational private book market.

### Legal regulations of language policy and curriculum development in South Africa

Colonialism, migration of different African groups to what is today South Africa as well as the apartheid regime shaped and influenced languages, language attitudes and language policies. The Bantu Education Act of 1953 and the Soweto Uprising of 1976 are only two examples indicating the emotional and conflict-laden language policy during that time. The key feature of the Bantu Education Act of 1953 was the focus on mother tongue education with its simplified curriculum for black pupils (Hurst, 2016, p. 222), a clear ‘inferior and humiliating curriculum’ for black pupils (Alexander 2003, p. 14). Such aimed at the racial or ethnic grouping of African people and the segregation of black people into Bantustans (Plüddemann, 1999, p. 327). The entire education policy starting from the 1950s in the apartheid South Africa aimed to establish 'linguistically zoned' (Kamwendo, 2006, p. 56) areas and ethnically separated curricula (Thobejane 2005, p. 3; Kamwangamalu 2001, p. 391). Overall such segregated education systems prepared each racially constructed group for their specific designated and limited role in society and therefore limited access to English and Afrikaans, the two official languages of the country at that time. Such affected the corpus planning for African languages negatively, as it was limited to their restricted usage at primary schools (Kros 2010, p. 111).

„English and Afrikaans were compulsory subjects for matriculation for all students, and African languages had a subordinate position in the apartheid education system. The situation in schools mirrored the situation in the broader society” (Gilmartin 2004, pp. 406–407).

During apartheid, no overarching curriculum was in place due to the separation of the system, but rather different ones for each racial designated education system. All African languages had a common curriculum differing from English and Afrikaans. Only in 1989 different curricula were introduced for all African languages except for lesser spoken African languages such as isiNdebele and siSwati (Murray 2012, p. 87). Therefore language policy represented the apartheid regime and became the centre of opposition. The peak of such culminated during the June 1976 Soweto Uprising where hundreds of pupils were slaughtered as a reaction to the plan to introduce Afrikaans as LoLT at the so-called Department of Education and Training schools (DET), which were designated for black pupils.

„The Soweto uprising was a culminating point in South Africa's long history of segregated and unequal education, which was the norm from the earliest days of British rule" (Fiske and Ladd 2004, p. 41).

Language policy was highly racial and the status of Afrikaans caused many conflicts. Afrikaans was interpreted and seen as the language and representation of apartheid (Kamwendo 2006, p. 56; Brock-Utne und Holmarsdottir 2004, p. 72). Using African languages as LoLT was highly stigmatized and ‘became synonymous with inferior education’ (Kamwangamalu 2019, p. 51). To overcome such a historical legacy and to reconcile the country, the Post-apartheid government finally decided to declare eleven official languages.

Art. 29 defines education and speaks also about language policy and uses terms such as ‘practicability’ and ‘reasonable educational alternatives’ to somehow limit the very progressive multilingual tone of this article and the Constitution in general.

„29. Education

[…]

(2) Everyone has the right to receive education in the official language or languages of their choice in public educational institutions where that education is reasonably practicable“ (Republic of South Africa 1996, Art. 29).

Other very important legal regulations are the National Education Policy Act (NEPA) of 1996, the South African Schools Act (SASA) of 1996 and the Language in Education Policy (LiEP) of 1997. In addition, multiple policy documents were published in the last decade regarding various aspects of education. Compared to the situation in Kenya, the language policy in South Africa was much more consistent, at least from a legal perspective. Since Post-Apartheid the LiEP remained in place and was not modified. Hence, the macro-level language policy regulations were unchanged for decades. Due to its main focus on curriculum developments, the Incremental Introduction of African Languages (IIAL) policy document was an initiative to boost substantially the use of African languages at schools. Its aim was that every pupil in South Africa should learn, at least to some extent, an African language. Such clear legal favour of multilingualism and African language usage is far less clear in Kenya. In addition on a macro-level, no single African language will be favoured over any other African language. As will be shown below on a micro-level each school can define their language policy precisely. Furthermore, other stakeholders influence the South African education system. Very important and influential in the South African context are the various trade unions as nearly all teachers are members of trade unions. Hence, bottom-up initiatives and influences appear quite frequently in South Africa. All in all, the South African education system reflects the existing social inequalities so some authors speak of a bimodal education system with no existing 'average pupil’ or school (Spaull 2013, pp. 440–444).

In Post-apartheid South Africa, the first curricular reforms tried to abolish all traces of its apartheid history (Spaull 2013, p. 436). This included the abolishment of several education ministries to one national and nine provincial education ministries. The provincial ministries' responsibility is the implementation of the one national curriculum (Chisholm 2012, pp. 91–92). Following global processes of devolution, CCP and CBC the SASA offered schools several rights. Hence, schools formulate their language policy on a micro-level and decide what language(s) will be selected as LoLT or so-called Home Language (HL) or other subjects. In general, the School Governing Body (SGB) gave schools an increasingly autonomous function. Parents or legal guardians represent over 50% of the SGB (Department of Education 1996). This led to a socio-economic bias as only parents in a good socio-economic situation are members of the SGBs, which are also often in favour of English. Due to wrong perceptions about languages a biased positive language attitude towards English and an unreasonable negative language attitude towards African languages regardless of the existing research, a majority of parents or legal guardians are in favour of English-medium schools. Ndhlovu (2015, p. 171) highlights that the underlying reason is the better equipped English-medium schools compared to African-Language-medium schools. The LiEP is one main language policy document. Unfortunately, the various curricula changes were never linked to the LiEP, so these reforms never allowed a coherent language policy (Heugh 2002, p. 466). In general, the LiEP aims to strengthen the African languages and foster multilingualism. The Department of Basic Education only in 2007 systematically researched to get an overview of the selected languages at South African schools. The report indicated a decline in English and an increase in isiZulu between 1998 and 2007 so in Grade 1 isiZulu was the main LoLT (DBE 2010). Nevertheless, the implementation of the LiEP differs widely within South Africa. Research by Kretzer (2018) showed that school language policy documents exist on a continuum. Some have very detailed and free language policy documents, which included forms of CS while others used only a template of the DBE (Kretzer and Kaschula 2020) as shown for schools in Limpopo. Contrary to Kenya, South Africa changed or rather revised its curricula on a rather frequent basis, whereas Kenya revised their language policy rather frequently.

In 1998 South Africa introduced the so-called C2005 curriculum to all grade 1 s and aimed to roll it out in phases until 2005. The implementation of the C2005 must be seen in the historical context of apartheid and despite its original ideas coming from outside of South Africa; it reflected and offered so much due to its maximum distance or opposition to education during apartheid. Chisholm puts it so rightly: 'Regardless of its being a borrowed set of ideas, then, outcomes-based education had become imbued with local content – it signalled the move from apartheid curricula (Chisholm 2005, p. 91). Within the C2005 only one out of the eight learning areas, namely ‘Language, Literacy and Communication’ actually mainstreamed the aspect of language for the curriculum. Therefore Plüddemann is so right when he mentioned that ‘Curriculum 2005 is flawed in tacitly assuming an English-only approach’ or when he states ‘that the role of language in concept formation has not been given sufficient thought’(Plüddemann 1999, pp. 332–332).

The influence during the C2005 development from various internationally, mainly Anglophone experts was a major shift for all subjects, but especially for African languages and to a lesser extent for Afrikaans and much lesser to English due to two reasons. Firstly, the used Anglocentric worldview and elements such as critical literacies were much more common for teachers of English compared to rural African language teachers. Secondly, the path dependencies of the apartheid regime remained. Already in 1973, the English curriculum included a personal growth approach and free expression for the pupils, whereas Afrikaans and even more African languages focused predominantly, if not exclusively, on grammar and phonology (Murray 2012, p. 87).

Jansen (1998, p. 321) defined this reform as 'the most ambitious curriculum policy'. C2005 was developed by Outcomes-Based Education (OBE)-curricula concepts. This includes the main focus shift towards pupils-centred lessons and less focus on specific content, but rather on learning and training of so-called outcomes or competencies. The idea originated in the USA and highly influences education systems globally (Chisholm 2015, p. 401). The pupil-centred approach led to more group work and more pupil-to-pupil communication, which was done mainly in indigenous languages. Teachers did not receive any help regarding how to deal with it or language policy in general (Niedrig 2004, p. 89). Teacher training was even more important with this curriculum reform because the focus shifted from teacher to pupils and changed the way of teaching and the teachers' role. It included group and partner work instead of teacher-dominated lessons on the blackboard. C2005 aimed at ongoing assessments in various forms as well as a much stronger focus on literacy and numeracy. Therefore, language policy and language-related challenges were supposed to be at the core of C2005, but unfortunately, this was never the case.

South Africa undertook several curricular reforms and modifications of its C2005 curriculum due to protests from teachers and unsatisfactory results from various national and international evaluation studies. In 2002, South Africa modified the C2005^2^ curriculum to the Revised National Curriculum Statement (RNCS). Many teachers saw the C2005 and the RNCS as two distinct curricula, rather than the RNCS as a reform of the C2005 (Pudi 2006, p. 101). The core OBE elements of the C2005 are still retained in the RNCS, but curriculum materials were more precise (Junge 2017, p. 419; Chisholm 2015, p. 409). The RNCS was a hybrid curriculum between the C2005 and the traditional curriculum. It was published in all eleven official languages in 2002, but as Murray highlights rightly ‘conceptualized in English [and] the core document was translated or versioned into the other ten languages’ (Murray 2012, p. 88). Such an approach itself is already very problematic to use English as a blueprint for all other language curricula. All languages are different and have their cultural roots and path dependencies from apartheid. Using now English curriculum as the blueprint for all can only negatively impact African languages. Furthermore, from a linguistic perspective English, Afrikaans and African languages differ significantly orthographically and morphologically. The RNCS still used learning areas instead of subjects as a term. The RNCS tried to connect itself better to the LiEP with its additive multilingualism through the introduction of separate curricula for the language-related subjects, which were named: Home Language (HL), First Additional Language (FAL) and Second Additional Language (SAL). Like the previous curricula, the 2010 introduced CAPS ignored language policy. It was only mentioned regarding language subjects but never emphasized aspects about LoLT for all learning areas or grades. Instead, it remained vague and silent. With such inconsistent policies, it favoured an early-exit model for English (Plüddemann 2015, p. 190). Again the curricula for all languages were just versioned from English and a common framework existed for all eleven languages for the three language-related subjects or learning areas. Effects of word recognition accuracy as a measure for reading in HL subject might work well for English, but no specific measures were in place for the agglutinating African languages (Wildsmith-Cromarty & Balfoud 2019, p. 301).

A majority of teachers still struggled and complained about the new RNCS curriculum. One of the major challenges of the C2005 was its political dimension and that it was hardly a pedagogical, but a political project. South Africa aimed to have a curriculum like countries of the Global North, despite the fact, that such a 'resource hungry' curriculum can hardly be implemented smoothly in an under-resourced education system like South Africa. In the Curriculum Review of 2009 teachers saw the RNCS as ‘burdensome’ and were in favour of 'proper examinations' (Department of Basic Education 2009, p. 35). Therefore, starting in 2010 South Africa introduced the CAPS with clearly defined learning targets for subjects and terms (Chisholm 2015, p. 411; Department of Basic Education 2011). The main aim was to support schools in severe socio-economic areas with challenging and very difficult learning environments. Therefore, tests and various examinations are very detailed and content-defined and described (Kanjee and Sayed 2013, p. 460). CAPS can hardly be seen as a CBC curriculum.

Some very promising developments happened recently regarding African languages. First, 90 pilot schools started offering Kiswahili as a subject as the first Non-South African, African language in 2019 and it is planned to further expand it (Xinhuanet 2019). Second, the Mother Tongue-Based Bilingual Education (MTBBE), which was pioneered in Eastern Cape is planned to be extended to other South African provinces. After a pilot phase, it was implemented at ten schools in all education districts in Eastern Cape in 2017 and increased in 2018 to 50 schools per district. Pupils are taught in English and isiXhosa to overcome language-related challenges (Mail and Guardian 2018). Third, the success of the MTBBE, where now over 2,000 schools use isiXhosa and Sesotho as LoLT beyond grade 3, motivated the education ministry to think about substantial language policy revisions. The final examinations of those cohorts in Eastern Cape, who participated in the pilot phase as grade 4 pupils in 2012 outperform English-medium school pupils. Hence, the DBE plans to change the LoLT at final assessments to include African languages (Business Tech 2020). Fourth, in 2020 Stellenbosch University introduced a new module called 'Multilingual Education'. It was the first time such a module was introduced at the Bachelor of Education level to teach language education more holistically and not only in terms of separate language subjects (van der Walt 2021, p. 7). Those examples are promising, but also indicate how fragmented language policy appears in South Africa, which is a chance and risk at the same time for African languages. Such developments and fragmented, if not isolated initiatives are quite common for language policy in African countries and some above-mentioned patterns appear in Kenya in a very similar way.

### Kenya’s educational commissions, its curricula and language policy changes

Kenya's language policy is influenced during colonial times by various actors. The colonial administration, Christian missionaries and European settlers tried to direct and influence colonial language policy. Therefore, the status of English and the African languages differed as no coherent language policy existed (Wanjiku-Omollo 2014), but rather conflicting positions regarding the status of English, Kiswahili and the indigenous languages (Kibui 2014, p. 90). Unlike in South Africa, one African language, namely Kiswahili is in quite a special position and excels the other African languages in Kenya. No African language in South Africa has such a dominant position, at least not on the national scale.

In 1909 the Christian missionaries were in favour of using Kiswahili at the beginning of primary schools as LoLT. Like in South Africa the missionary societies' influence on indigenous languages was twofold due to the role they played regarding orthography development for African languages, but also their arbitrary selection or separation of language varieties. The Phelps-Stokes-Commission favoured in 1924 the usage of the local dominant indigenous languages for the first years at primary schools at the expense of Kiswahili, which was limited to its geographical stronghold. Such limitation policy aimed at counteracting Kenyan liberation initiatives, which were often related to Kiswahili (Njoroge and Gathigia 2017, p. 77; Campbell and Walsh 2009, p. 581). Nevertheless, only four African languages were recommended for use at schools, namely Kiswahili, Dholuo, Luhya and Gikuyu. At a later stage, Nandi was added. Such eclectic, inconsistent and often changing language policy was one of the main characteristics during colonial times. The Inter-Territorial Language Committee in 1930 strengthened Kiswahili again when the Zanzibar Kiswahili variety was selected and standardized. The decade of colonial government up until the Second World War was described by Nabea:

Contrary to the long-held postulation that it was the objective of the colonial government to promote the English language in the colony, the colonial language policy was always inchoate and vacillating such that there were occasions when measures were put in place to promote or deter its learning. (Nabea 2009, p. 122).

It was motivated to ensure the social distance between colonizers and colonized through linguistic distance. This explains the stronger focus on indigenous languages to weaken Kiswahili. Education was used to establish a 'buffer zone' between English, Kiswahili and the other indigenous languages (Campbell and Walsh 2009, p. 581).

The decades following the Second World War were characterized by a stronger focus on English. Such language policy had a long-lasting influence on language attitudes, which of course are also shaped by globalization or media. The idea or belief that education and development can only be achieved through inherited colonial languages is deeply rooted and defines African languages as incapable of doing so (Chimbutane 2017, p. 356). Three reports, namely Beecher (1949), Binn (1952) and Drogheda (1952) aimed for a stronger focus on English and thus ultimately for a stronger ‘Westernization’ of the Kenyan elite (Njoroge and Gathigia 2017, p. 77). Hence, the status of Kiswahili was hereby mainly addressed and weakened (Nabea 2009, p. 124), because a trilingual language policy at primary schools was seen as inappropriate. In 1953 Kiswahili was even banned by the colonial government (Mbithi 2014, p. 5). Interestingly enough the Beecher report suggested the use of eight African languages in addition to Kiswahili. Those languages were Kidawida, Kikamba, Gikuyu, Maasai, Kimeru, Nandi (for Kalenjin languages), Oluluyia and Dholuo (Mwaniki 2014, p. 11). Despite the recommendation of those three commissions to use African languages it hardly strengthened those languages as their usage was limited to lower grades.

With the independence of Kenya in 1963, English became the official language. The Ominde Commission of 1964 followed the previous mindset accordingly, but (re)introduced Kiswahili in primary schools. The New Primary Approach also known as the English-Medium Approach recommended the use of English from Grade 1 as LoLT (Mose and Kaschula 2019, p. 331; Nabea 2009, p. 125; Ogechi 2009, p. 147). From 1967 onwards the Kenyan Institute of Education (KIE) started working on primary school textbooks in African languages. In 1968 the Tujifunze *Kusoma Kikwetu*—Let us learn our mother tongue (TKK) textbooks were published as a series in 15 African languages (Mwaniki 2014, p. 11). It remained an unchanged assumption that larger African languages represent smaller related African languages. Hence, no need was seen to produce materials in all indigenous languages for the TKK series. Today the series includes 22 African languages (Kibui 2014, p. 92), which is a quite multilingual approach and influenced corpus planning of indigenous languages in Kenya broadly by incorporating such a high number of African languages in the TKK series. Although those African languages were not official, it is clear that language policy is not limited to English and Kiswahili only, but includes many other indigenous languages as well. This is quite similar to South Africa but without the status planning and declaration of eleven official languages. A change of language policy occurred with the Gachathi Commission in 1976. Its recommendations focused on a three-year usage of African Languages as LoLT and made Kiswahili an examination subject at the end of primary school. The usage of African languages was limited to rural linguistically homogenous areas and the inferior status of Kiswahili remained and became visible through a smaller allocation of hours compared with English. A clear trilingual system was in place. Because Kenya is predominantly a rural country with its citizens living in linguistically rather homogenous communities, this policy favoured African languages. Only a few areas are urban or semi-urban so only there Kiswahili was LoLT according to the language policy. Some officials and/or teachers struggled to define urban, semi-urban or rural settlements and therefore did not use African languages accordingly. Hence, many schools used English as LoLT even from grade 1 onwards despite the official language policy (Ogechi 2009, p. 147), sometimes simply because those language policy documents do not define a specific language for every specific county (Mandillah 2019, p. 8). In general, previous experiences in Kenya indicated a lack of commitment regarding the development of materials in African languages, so that they can be used in schools. Some authors even questioned whether the Kenyan government is in favour of such a language policy or if it is not only lip service for African languages as the lack of commitment is overwhelming. An example is the Koech Commission of 1999 and the rejection of its recommendations raised again general questions about Kenya’s position on research studies and education planning (Njoroge and Gathigia 2017, p. 79). Many authors criticize in general the top-down strategy, which is often applied by the Kenyan government (Muricho and Chang’ach 2013, p. 125), which contrasts with the situation in Post-apartheid South Africa. Teacher unions and other civil society actors were much more involved during the drafting of such policies.

Certainly, there is no ready evidence of state-initiated support for teacher training, local-language materials development or advocacy with educators and community leaders on behalf of the policy. Not only so, but the normalcy of resistance to the national language policy seems to indicate that local actors do not think the state is seriously behind it either (Trudell and Piper 2014, p. 17).

This language policy approach remained unchanged until recently and was only sometimes re-instated through directives such as the Sessional Paper Number 14 of 2012 (Academia Kenya 2012).

The 8–4-4 structure of the Kenyan education system was formulated by the Mackay Commission in 1981. This commission made Kiswahili a compulsory and examinable subject for primary and secondary schools (Mbithi 2014, p. 5). The eight years of primary school, followed by four years of secondary school and four years at the university level remained in place until the full implementation of the CBC in 2019.

A report by Wamalwa in 1972 introduced French and German to the secondary school syllabus as two ‘foreign’ languages. The Odhiambo Task Force of 2012 brought the introduction of Mandarin as a subject in Kenya into play, which is similar to South Africa. Mandarin is also offered as a school subject in South Africa.

In general, in Post-independence Kenya English was favoured at schools. This was in reality even stronger than already stipulated within the numerous language policy documents. Pupils encounter humiliating experiences at school if they spoke their indigenous languages outside of the designated time and space (Wanjiku-Omollo 2014, p. 16). They were forced to wear a plate with statements such as ‘I am a donkey’ (Spernes and Ruto-Korir 2018, p. 51; Nabea 2009, p. 126). Such experiences increased the already existing division between English and indigenous languages and created an alienation of Kenyan pupils from their languages, cultures and communities.

### Implementation of the BECF

The previous curriculum was seen as no longer appropriate for the needs of the 21st century for the demands at workplaces, which 'produces' school graduates with a skill-set no longer needed at many workplaces and explains partly the high unemployment rates (KICD 2020; Republic of Kenya 2019a). Concerns were strong that it did not inculcate critical thinking skills e.g. reading entailed reading a passage followed by recalling 'wh- questions'. It was teacher-centred, with passive learning while the newly developed CBC is learner-centred. The previous curriculum was exam-oriented, whereas CBC focuses more on outcomes, experiences and ongoing assessments. The Kenya Institute of Curriculum Development (KICD) initiated 2009 an evaluation of the 8-4-4 education system and its curriculum, followed in 2016 by a Needs Assessment to find out what critics regarding the current curriculum exist. In 2017 the Basic Education Curriculum Framework (BECF) was approved, after several national stakeholder conferences (KICD 2020). Such an approach was rather quite new, at least to that extent, and included in Kenya in a very similar way to South Africa now many different stakeholders during the drafting of the new curriculum. Before its full implementation, a 'tryout' was done in 2017. 470 schools, 10 from each county were selected to start a trial implementation of the new CBC (KICD 2020).

In addition, several thousand teachers received training on the new curriculum and ongoing monitoring of its implementation was established (KICD 2020).

The CBC is meant to equip learners with 21^st^-century skills needed to produce multi-skilled pupils with relevant competencies for work and life. CBC emphasizes becoming ethical citizens through a value-based approach to education (UNESCO 2018b, p. 30). Parents have also involved in teaching their children. Hence, parents, legal guardians or the local community can support and be better involved in pupils' education if a familiar African language has been used (Kioko et. al. 2014, p. 6).

The 8-4-4 system focused on lectures and theory, whereas the CBC is more ‘Hands-on’ (KICD 2020). Structurally, the 8-4-4 will be transformed into a 2-6-3-3 education system, covering 2 years of early year education or pre-primary, followed by 6 years primary, 3 years junior secondary and 3 years of secondary school. Learning is supposed to be experiential and practical through field trips and various internship programmes with companies. The previous curriculum taught how to plant a tree theoretically and teacher-centred on a blackboard with maybe a picture or drawing to illustrate, while the new CBC aims at planting such tree by pupils. Such a shift is directly linked to replacing the previous focus on memorizing content during lessons with a more interactive way of teaching. The example of planting a tree indicated such and can be done through a team project and peer learning so that the social structure and role of pupils and teachers need to be significantly revised and changed.

Hence, KICD (2020) highlights it was necessary to transform the education system and link all subjects to four core skills (‘4cs’). Interestingly enough the first ‘C’ (‘Communication and collaboration’) mentions various languages, but only Kiswahili as an African language and no other indigenous Kenyan language. Subjects or Learning Areas like Kiswahili, English, Kenyan Sign language or foreign languages (Arabic, French, German or Mandarin) are grouped (Republic of Kenya 2019b, c). None of the other African languages is directly named here to clarify its usage. The same repeats in the ‘Orange book’, a list of accredited textbooks for schools. English and Kiswahili are named, but other African languages are only referred to as 'mother tongue' and their materials are listed (KICD 2017a; b).

Overall the BECF mentions ‘language’ 149 times, English 31 and Kiswahili 32 times. Contrary indigenous languages are only mentioned 17 times and never any indigenous languages are directly named. Only few terms are translated into Kiswahili, such as *Lugha ya Kiswahili* and *Fasihi ya Kiswahili* (KICD 2017a; b, p. 61). Similarly to the South African curricula the BECF was also exclusively conceptualized in English. The Grade 5 CBC material for language learning areas like German and French entails some German and French words to elaborate on what competencies should be taught. The learning area of indigenous languages is vague and does not entail any non-English words, because it is used as an umbrella for all indigenous languages, which is fraudulent from the beginning (KICD 2019). If all those indigenous African languages would be interchangeable, then ONE learning area for ALL literacy-related competencies would be enough. It would include English, Kiswahili and all foreign languages as well as the other African languages of Kenya. The way the Grade 5 CBC material is described, reflects again a devalued view of African languages, as they are presented as inferior compared to other literacy learning areas.

## Implementation challenges of pupil-centred curricula reforms in Kenya and South Africa

Although both countries tried to include teachers to varying degrees during the conceptualisation or at least during the piloting of the new curricula still many struggled or even resisted the new curriculum, whereas others implemented or modified their teaching accordingly. Earlier studies done by the authors revealed interesting insight into the Linguistic Landscapes at South African schools or language attitudes and perceptions by principals.

The Linguistic Landscape (LL) showed for South African schools a huge variety of information leaflets either multilingual in Afrikaans or English or one African language or even multilingual in two or more languages (Kretzer and Kaschula 2019). One principal from a township school in Gauteng tried to be as inclusive as possible and asked pupils about their parents' language preferences and produced information materials accordingly.

Look when you communicate with parents we try to have the communiqué written in different languages so that they can be in the position to understand what we say. […] From learner's preference of parents then they will tell us to say no my mum or my dad wants this circular to be in this and then we call in that information so that when we make circulars we know so many must be English, so many in Zulu, so many in Sotho and then we distribute them to learners to parents (Kretzer 2018, pp. 225–226).

Contrary, a study was done by Spernes and Ruto-Korir (2018) in Nandi County in Kenya showed the influence of language attitudes. This area was a rural site so the language of the catchment area is Nandi. Therefore, Nandi was supposed to be the LoLT. Interestingly the majority of schools did not use Nandi due to their language attitude and their perceptions of the parents' language attitudes.

Interviewed principals indicated retrospectively about the C2005 their scepticism and saw the OBE as a pure ‘First-World’ project, which was not appropriated or adjusted to the situation at South African schools.

We switched over to OBE, we weren't ready for OBE, we weren't, our teachers were not qualified enough, and we didn't get enough training for OBE. We were, we are third country, […] we were compared with Australia, England and staff […] In our country, you can't compare us with them, because we didn't have the knowledge, our learners were not exposed, the little ones you know before coming to school they were not exposed to like first world countries. They haven't attended crèche […] they didn't have the stimulation and stuff like that, they should have had, so that, that was to me … a problem (Kretzer 2018, p. 198).

In relationship to potential adjustments or modifications of language policy at schools studies in South Africa and Kenya showed different reactions.

An earlier study done by Mose and Kaschula (2019) indicated a lot of CS happening in Gusii in western Kenya. Ekegusii is used in non-examinable subjects as well as to try to explain previously taught concepts in English again in Ekegusii to ensure pupils understood it. Teachers seem to be indecisive on how to teach pupils right in Kenya. Mandillah (2019, p. 11) sees CS with greater variety, as she includes hybrid codes such as Sheng during a teaching in Kenya. Likely, Sheng is quite frequently used by pupils (and teachers) during lessons in urban or semi-urban contexts, taking into consideration its long history and its broad social use.

This situation is partly different in South Africa due to the micro language planning layer. Unlike in Kenya schools have the right and duty to formulate their school language policy. Some have very open, flexible and multilingual language policy documents and even allow CS during lessons (Kretzer and Kaschula 2020). Although this is the exception, because the vast majority of schools formulated either vague or very strict language policy documents, such progressive schools existed. Within the South African context, CS is quite widespread, so in the Bojanala district of North West Province, Setswana is used throughout all grades more often than English for oral communication. This is quite surprising and is not reflected in the official school documents. Although Setswana is mentioned often in primary school language policy documents and also often used in information leaflets, in reality, Setswana is even more often been spoken during lessons. It is often the second most used language for the three research provinces due to its dominance in North West (Kretzer 2018).

As indicated above with the example of Uganda similar behaviour pattern occurred in Kenya. A very recent study by Mandillah (2019) done in Bungoma County in the western area of Kenya showed similar trends to the results from Uganda previously shown. Teachers tend to revert to traditional ways of teaching, including a very teacher-centred lesson with very limited interaction between pupils and their role was again mainly limited to choir answers or repeating content.

## Conclusion

Regardless of those language policy documents on a national (macro), provincial or county (meso) or even school level (micro), ultimately teachers implement fully, partly or reject any official language policy. Such depends heavily on their ‘buy-in’ of the new curricula or general educational reform or language policy. Therefore, teachers need to be involved as much and as early as possible in the development of language policy and curriculum reforms. The example of South Africa and its failed C2005 curriculum reflects this, regardless of whether those teachers were heard before its implementation. They felt not included and were overwhelmed by the massive educational changes in South Africa. Only if teachers are involved in a real meaningful way will there be a chance of a successful implementation. Furthermore, any child-centred pedagogy or competency-based curriculum will only work meaningfully if the language policy is coherent. This means any pedagogy being rooted in constructivism needs to use a language pupils are familiar with. Such pedagogy and curriculum should go hand in hand with a language policy favouring African languages as LoLT throughout. Through increased use of African languages, a curriculum itself can also be de-colonized and revised. Such an ‘Africanized’ curriculum taught through African languages of a certain area can finally ensure real meaningful education for the Kenyan and South African classrooms. Nevertheless, no one size fits all solution exists, but rather micro-level solutions similar to South Africa might help Kenya. Furthermore, Kenya had the chance to learn from the hasty and ultimately failed implementation of the C2005 in South Africa. Historical developments and socio-cultural conditions must be taken into consideration before such a massive education turnaround. A CBC and pupil-centred approach with its CCP offer in both countries widely unused potentials. The implementation in Kenya is too new to judge its ‘success’, but many obstacles exist in the formulation of its policy documents. The devalued way African languages are treated in the CBC materials for learning areas will likely be reflected in its implementation. The general political will of the Kenyan government for a real multilingual language policy at schools with its promotion of African languages is doubted. The clear triglossic situation between the very dominant English, the advanced Kiswahili and the hard or very limited used African languages hamper a real inclusive multilingual language policy and the meaningful implementation of the BECF.

The situation looks contrary in South Africa. Although the CBC-based C2005 curriculum and its reformed version failed substantially and African languages were also not promoted strongly, recent developments give some hope. In addition, nine African languages are official and treated much more equal compared to Kiswahili and especially the other African languages in Kenya. A clear English dominance prevailed in South Africa, too. Additionally, the pilot phase for Kiswahili as a subject might be only a small, but meaningful decision in favour of African languages for the whole African continent. More important is the success of the MTBBE programme and statements of the education ministry in mid-2020 regarding substantial language policy changes. Nevertheless, African languages play still a minor role in both countries and the language curricula of African languages must be analysed more in detail. In general, both countries reflect that language policy and curriculum developments were never coherent and made both its successful implementation and meaningful teaching through African languages for the majority of pupils difficult if not impossible.

1. Terms such as Francophone, Anglophone or Lusophone are labels from the Global North, have their origins in colonial times and do not reflect the complex linguistic situation. Due to the fact, that these categories are commonly known they are also used here and do not have any political message.
2. C2005 or OBE and NCS are all the same curriculum and just three names for it.
